# SQLE facilitates the pancreatic cancer progression via the lncRNA‐TTN‐AS1/miR‐133b/SQLE axis

**DOI:** 10.1111/jcmm.17347

**Published:** 2022-05-31

**Authors:** Shuhui Wang, Lei Dong, Lin Ma, Suzhen Yang, Ying Zheng, Jing Zhang, Chuanghong Wu, Yidi Zhao, Yangfan Hou, Hong Li, Ting Wang

**Affiliations:** ^1^ Department of Digestive Disease and Gastrointestinal Motility Research Room The Second Affiliated Hospital of Xi’an Jiaotong University Xi’an China; ^2^ Department of Infectious Diseases Shenzhen Nanshan District Shekou People’s Hospital Shenzhen China; ^3^ Department of Gastroenterology Shaanxi Provincial People’s Hospital Xi’an China; ^4^ Department of Kidney Transplantation Nephropathy Hospital The First Affiliated Hospital of Xi'an Jiaotong University Xi’an China; ^5^ Emergency Department The Second Affiliated Hospital of Xi’an Jiaotong University Xi’an China; ^6^ Department of Respiratory and Critical Care Medicine The Second Affiliated Hospital of Xi’an Jiaotong University Xi’an China

**Keywords:** lncRNA‐TTN‐AS1, miR‐133b, pancreatic cancer, SQLE, terbinafine

## Abstract

Studies have shown that SQLE is highly expressed in a variety of tumours and promotes tumour progression. However, the role of SQLE in pancreatic cancer (PC) has not been reported. Here, we aim to study the role and molecular mechanism of SQLE in PC. Immunohistochemistry and functional experiments showed that SQLE was highly expressed in PC tissues and promoted the proliferation and invasion of PC cells. Terbinafine, an inhibitor of SQLE, inhibited this effect. In order to further study the upstream mechanism that regulates SQLE, we used bioinformatics technology to lock miR‐133b and lncRNA‐TTN‐AS. In situ hybridization was used to detect the expression of miR‐133b and lncRNA‐TTN‐AS1 in PC tissues. The luciferase reporter gene experiment was used to confirm the binding of miR‐133b and lncRNA‐TTN‐AS1. The results showed that miR‐133b was down‐regulated in PC tissues and negatively correlated with the expression of SQLE. LncRNA‐TTN‐AS1 was upregulated in pancreatic cancer tissues and positively correlated with the expression of SQLE. Luciferase gene reporter gene analysis confirmed lncRNA‐TTN‐AS1 directly binded to miR‐133b. Therefore, we propose that targeting the lncRNA‐TTN‐AS1/miR‐133b/SQLE axis is expected to provide new ideas for the clinical treatment of PC patients.

## 1 INTRODUCTION

Pancreatic cancer (PC) is one of the deadliest cancers in the world. Based on GLOBOCAN 2020 estimates, PC is the seventh leading cause of cancer death in both males and females.^1^ Despite ongoing developments, PC remains one of the most difficult tumours to treat, and the five‐year survival rate is <11%.^2^ Therefore, it is of great significance to explore the molecular mechanism of PC progression and find new biomarkers and targeted therapies.

Squalene epoxidase (SQLE), found on chromosome 8q24.13 in humans,^3^ catalyzes the stereospecific conversion of squalene to 2,3(S)‐oxidosqualene.^4^ It is a key step in cholesterol biosynthesis and has been proposed as a second rate‐limiting enzyme in cholesterol synthesis.^5^ Studies have reported that SQLE is overexpressed in many malignant tumours.^6^, ^7^, ^8^, ^9^, ^10^ Terbinafine is a drug that targets SQLE. A study has shown that it can inhibit the development of non‐alcoholic fatty liver disease‐related hepatocellular carcinoma.^11^ However, the role of SQLE in the development of PC is unclear.

MicroRNAs (miRNAs) are a class of non‐coding RNA molecules that play a central part in cell differentiation, proliferation and survival by binding to complementary target mRNAs, leading to mRNA translational inhibition or degradation.^12^ MiR‐133b is generally considered to be a muscle‐specific miRNA.^13^ Recent reports indicated that miR‐133b was dysregulated in a variety of cancers and leaded to malignant progression.^14^, ^15^, ^16^ Notably, the public database TargetScan indicated that miR‐133b may interact with SQLE, which suggested that miR‐133b may play a role in tumour progression by regulating SQLE. LncRNAs, as key elements of the competitive endogenous RNA (ceRNA) network, can be combined with miRNA to neutralize the inhibitory effect of target genes, thereby participating in the development of tumours.^17^ TTN‐AS1 is a lncRNA that binds to titin mRNA (TTN) and has pro‐oncogenic effects in many cancers.^18^ Increasing studies have reported the upregulation and oncogenic function of TTN‐AS1 in multiple cancer types.^19^, ^20^, ^21^ It was worth mentioning that the prediction of LncBase indicated that lncRNA‐TTN‐AS1 may be lncRNA targeting miR‐133b. These data indicated that lncRNA‐TTN‐AS1 was likely to target miR‐133b to regulate the expression of SQLE. However, the role of miR‐133b and lncRNA‐TTN‐AS1 in PC remains unknown.

Here, we explored the role of lncRNA‐TTN‐AS1/miR‐133b/SQLE axis in the development of PC. LncRNA‐TTN‐AS1 functioned as a sponge of miR‐133b to upregulate the expression of SQLE, thereby promoting the development of pancreatic cancer. Targeting the lncRNA‐TTN‐AS1/miR‐133b/SQLE axis is expected to provide new ideas for the clinical treatment of PC patients.

## 2 MATERIALS AND METHODS

### 2.1 Tissue microarray

Three PC tissue microarrays were purchased from Outdo Biotech Co., Ltd (Shanghai, China). Specimens of PC tissue and adjacent non‐malignant tissues were collected from patients who had undergone surgery. These tissues were from clinical phases 1, 2 and 4. The follow‐up time was November 2014. The interval was 1.2–5.8 years.

### 2.2 Immunohistochemistry

Paraffin‐embedded slides and tissue microarrays were dewaxed with dimethylbenzene and gradient alcohol. Citrate buffer was used for antigen retrieval. The slides were then incubated with 3% hydrogen peroxide for 15 min to inactivate endogenous peroxidase. Normal goat serum was added for 20 min at room temperature to block nonspecific staining. The slides were incubated with the primary antibody at 4°C overnight, then washed with phosphate‐buffered saline (PBS). Biotinylated secondary antibody was added and incubated at 37°C for 30 min. Next, the slides were washed with PBS and incubated with SABC for 30 min at 37°C. Then, stained with DAB and counterstained with haematoxylin. After washing with flowing water, the slides were dehydrated with gradient alcohol and dimethylbenzene. Finally, the slides were sealed with neutral balsam and coverslips.

### 2.3 Evaluation of immunostaining intensity

Two pathologists evaluated the degree of immunostaining by double blind method, and scored according to the percentage of positive cells and the intensity of staining. The percentage of positive cells was divided into five grades: no staining (0), 1–25% (1), 26–50% (2), 51–75% (3) and >75% (4). The intensity was scored as follows: negative (0), weak (1), moderate (2) and strong (3). A final histological overall score was calculated by multiplying the score for staining intensity with the score for percentage of positive cells. We regarded a score of 0–7 as low expression and a score of 8–12 as high expression.

### 2.4 Cell lines and transfection

Human PC cell lines CAPAN‐1, AsPC‐1 and the normal pancreatic ductal epithelial cell line HPDE were purchased from the Cell Bank of the Chinese Academy of Sciences. All cell lines have been identified by STR. SQLE overexpression and knockdown lentiviruses were bought from GeneChem Co., Ltd and performed according to the manufacture's protocol.

### 2.5 Cell proliferation and colony formation assay

The CCK‐8 assay was used to detect cell proliferation ability. The cells were seeded in a 96‐well plate at a density of 3 × 10^3^ cells with a volume of 100 μL. One plate was taken out every 24 h, and CCK‐8 reagent was added to each well. After reaction at 37°C for an hour, the absorbance of each well at 450 nm wavelength was measured by microplate reader. For colony formation assay, the cells were seeded in 6‐well plates with 1000 cells per well. After about 2 weeks of culture, the cells were fixed with methanol, stained with 0.1% crystal violet and counted.

### 2.6 Wound healing assay

The cells were seeded in the 6‐well plate. When the cells covered the bottom of the hole, they were scratched with a sterile gun. The cells were cultured in serum‐free medium and photographed.

### 2.7 Migration and invasion assays

For migration analysis, 200 μL serum‐free DMEM with 1.2 × 10^5^ cells were inoculated into the upper chamber. 650 μL DMEM with 30% FBS was added into the lower chamber. For the invasion assay, the serum‐free DMEM and Matrigel were diluted by 1:8 (operate at 4°C), and 60 μL was added into each well in the upper chamber. Then, dried in 37°C incubator for 6 h. 140 μL serum‐free DMEM with 1.2 × 10^5^ cells were inoculated into the upper chamber. 650 μL DMEM with 30%FBS was added into the lower chamber. After about 24 h of culture, gently wiped the cells on the surface of Matrigel and polycarbonate membrane with wet cotton swabs. Cells were fixed in methanol and stained with 0.1% crystal violet.

### 2.8 Mouse xenograft model

5‐week‐old male athymic nude mice were purchased from the Medical Experimental Animal Center of Xi'an Jiaotong University. All experiments were approved by Xi'an Jiaotong University. Each nude mouse was inoculated with 1 × 10^6^ cells subcutaneously, and the inoculation volume was 0.1 mL. After a month, the nude mice were sacrificed, the tumours were taken out, weighed and photographed.

### 2.9 Western blot analysis

Total proteins were extracted from the cells using RIPA buffer and a protease‐inhibitor cocktail. After electrophoresis in SDS‐PAGE, the proteins were transferred to PVDF membranes. The membranes were blocked with 10% milk and incubated with primary antibodies at 4°C overnight. The following antibodies were used: SQLE antibody (Abcam), c‐Myc antibody (Abcam), PCNA antibody (Abcam), CDK2 antibody (Abcam), cyclin D1 antibody (Abcam), β‐actin antibody (Abcam), GAPDH antibody (Abcam), p‐ERK1/2/ERK1/2 antibody (Abcam) and p‐p65/p65 antibody (Abcam). After washing with 1 × TBST for three times, the membrane was incubated with the corresponding secondary antibody.

### 2.10 Luciferase reporter assay

Luciferase activities were performed using the Dual‐Luciferase Reporter Assay System (Promega).

### 2.11 In situ hybridization

In situ hybridization (ISH) of lncRNA and miRNA with PC tissue microarrays were performed by Shanghai Outdo Biotech Co., Ltd. The sections were deparaffinized, digested by protease K, hydrated and deproteinated. About 20 μL of hybridization solution was then added to each slide. Each slide was covered with a coverslip and hybridized at 50°C for 1 h. The array slides were then rinsed and spin‐dried, placed on a humidified box at room temperature, dripped with 1 × blocking buffer and blocked for 15 min. Next, removed the blocking buffer on the slide, added 1:800 diluted anti‐DIG‐AP Fab fragments and stayed overnight at 4°C. The slides were then removed from the refrigerator, rewarmed at room temperature for 45 min and washed with PBST buffer. The working solution for colour development was configured according to the requirements of the BCIP/NBT Substrate kit instructions. PBST was used to clean the slides after colour development. Finally, the cells were counterstained with nuclear fast red for 3–5 minutes and mounted on slides.

### 2.12 Statistical analysis

SPSS statistics 18.0 and GraphPad Prism 8 were used for statistical analysis. The comparisons between two groups were performed using Student's *t* test. The chi‐squared test was used to determine whether there was a significant difference in the distribution of SQLE among the different categories. Survival curves were calculated using Kaplan–Meier and log‐rank tests. The effects of variables on survival were analysed by univariate and multivariate Cox proportional hazards modelling. Multiple group comparisons were analysed by one‐way ANOVA. Data were presented as means ± SD. All statistical tests were two‐sided, and values of *p *< 0.05 were considered significantly different.

## 3 RESULTS

### 3.1 SQLE is upregulated in PC and associated with a lower overall survival rate

**FIGURE 1:**
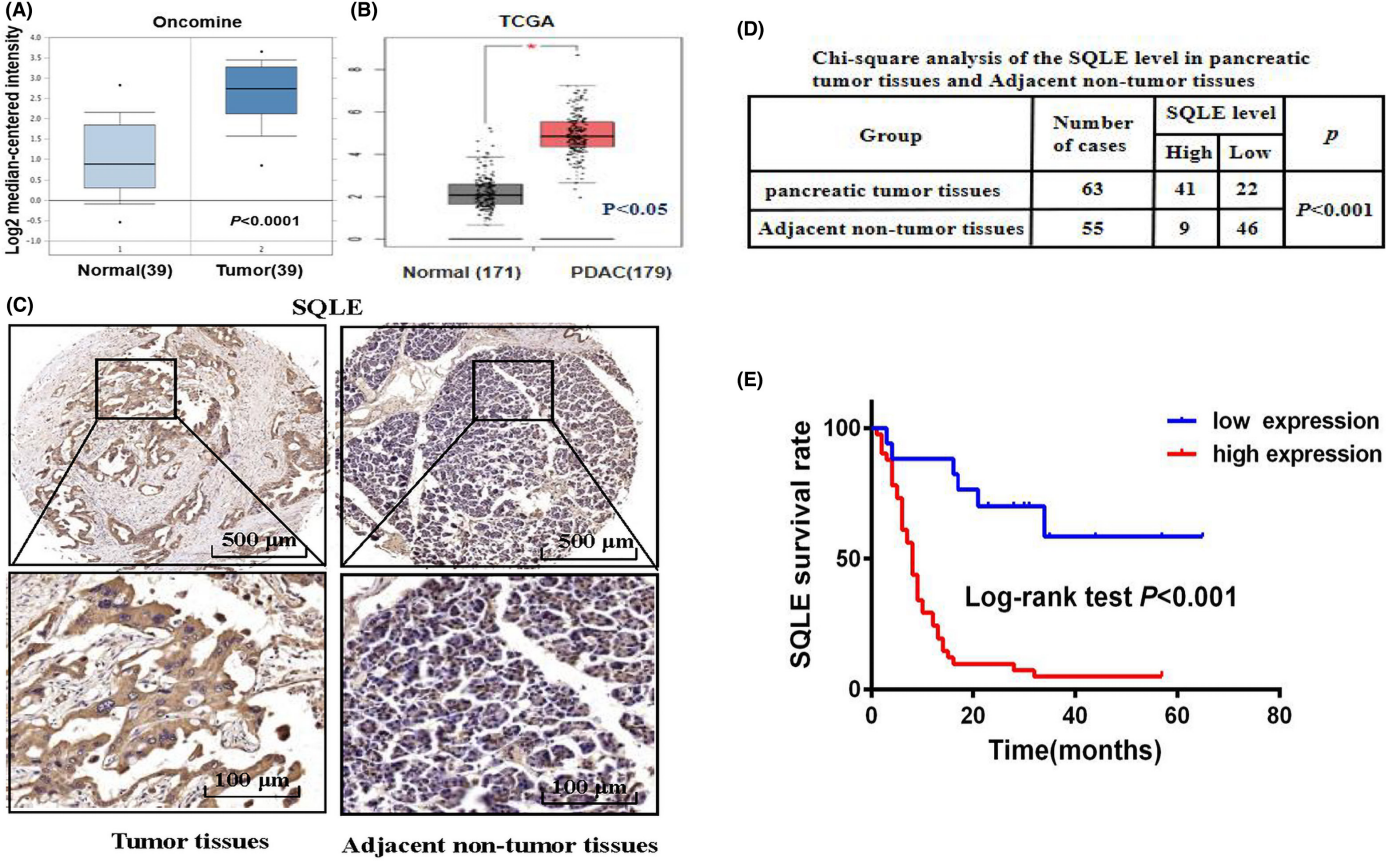
SQLE is upregulated in PC and associated with a lower overall survival rate. (A) The mRNA expression of SQLE in PC tissues and normal tissues in the Oncomine database. (B) The mRNA expression of SQLE in PC tissues and normal tissues in the TCGA database. (C) Representative images of IHC staining of SQLE in PC tissues and adjacent non‐tumour tissues. (D) Chi‐square analysis of SQLE levels in PC tissues and adjacent tissues. (E) Kaplan–Meier survival analysis of the overall survival in PC cases with high versus low SQLE expression

We analysed the mRNA expression of SQLE in PC tissues and normal tissues by Oncomine and TCGA databases. The results showed that compared with normal tissues, the mRNA expression level of SQLE in PC tissues was increased (Figure 1A, B). Next, we performed immunohistochemical analysis on the PC tissue microarray to detect the protein expression of SQLE and analyse the association between SQLE expression and prognosis of PC. The results showed that SQLE was highly expressed in PC tissues and lowly expressed in adjacent tissues (Figure 1C, D). Univariate analysis showed that distant metastasis, TNM staging and the expression level of SQLE were associated with a lower overall survival rate (Table 1). Further multivariate Cox regression analysis showed that SQLE was an independent factor in evaluating the prognosis of PC (Table 2). Moreover, PC patients with overexpression of SQLE had a lower overall survival rate and worse prognosis than those with low SQLE expression (Figure 1E).

**TABLE 1:** Prognostic factors in pancreatic cancer patients by univariate analysis

Variables	*N*	3‐year cumulative survival rate (%)	Mean survival time (month)	Risk ratio	95% confidence interval	*p*
Age (year)						
<60	21	17.0	13.5	1.566	0.767–3.201	0.218
≥60	42	17.0	8.7			
Sex						
Male	36	19.0	9.7	0.907	0.480–1.712	0.763
Female	27	13.0	13.5			
Tumor size (cm)						
<5	45	13.0	10.8	0.914	0.449–1.857	0.803
≥5	18	28.0	9.0			
Grade						
I–II	34	19.0	13.5	1.803	0.957–3.397	0.068
III	29	13.0	8.4			
T stage						
T1–T2	9	22.0	16.5	1.325	0.793–2.216	0.283
T3–T4	38	18.0	9.0			
Lymph note metastasis						
Negative	38	23.0	10.0	1.515	0.735–3.120	0.260
Positive	25	0.0	9.0			
M stage						
M0	59	18.0	10.8	13.841	2.243–85.407	<0.01
M1	4	0.0	5.0			
TNM stage						
I–II	50	21.0	14.0	0.278	0.079–0.984	<0.01
III–IV	4	0.0	5.0			
SQLE level						
Low	22	41.0	38.0	3.618	1.724–7.592	<0.01
High	41	4.0	8.5			

**TABLE 2:** Multivariate analysis using the Cox proportional hazards model

Variables	Risk ratio	95% confidence interval	*p*
M stage			
M0	0.335	0.105–1.064	0.064
M1			
TNM stage			
I–II	3.502	0.823–14.904	0.090
III–IV			
Grade			
I–II	0.602	0.322–1.127	0.113
III			
Tumor size (cm)			
<5	1.232	0.611–2.482	0.560
≥5			
Lymph node metastasis			
Negative	0.595	0.297–1.192	0.143
Positive			
T stage			
T1–T2	0.564	0.185–1.717	0.313
T3–T4			
SQLE level			
Low	0.259	0.122–0.548	<0.001
High			

### 3.2 SQLE promotes PC cells proliferation in vitro

**FIGURE 2:**
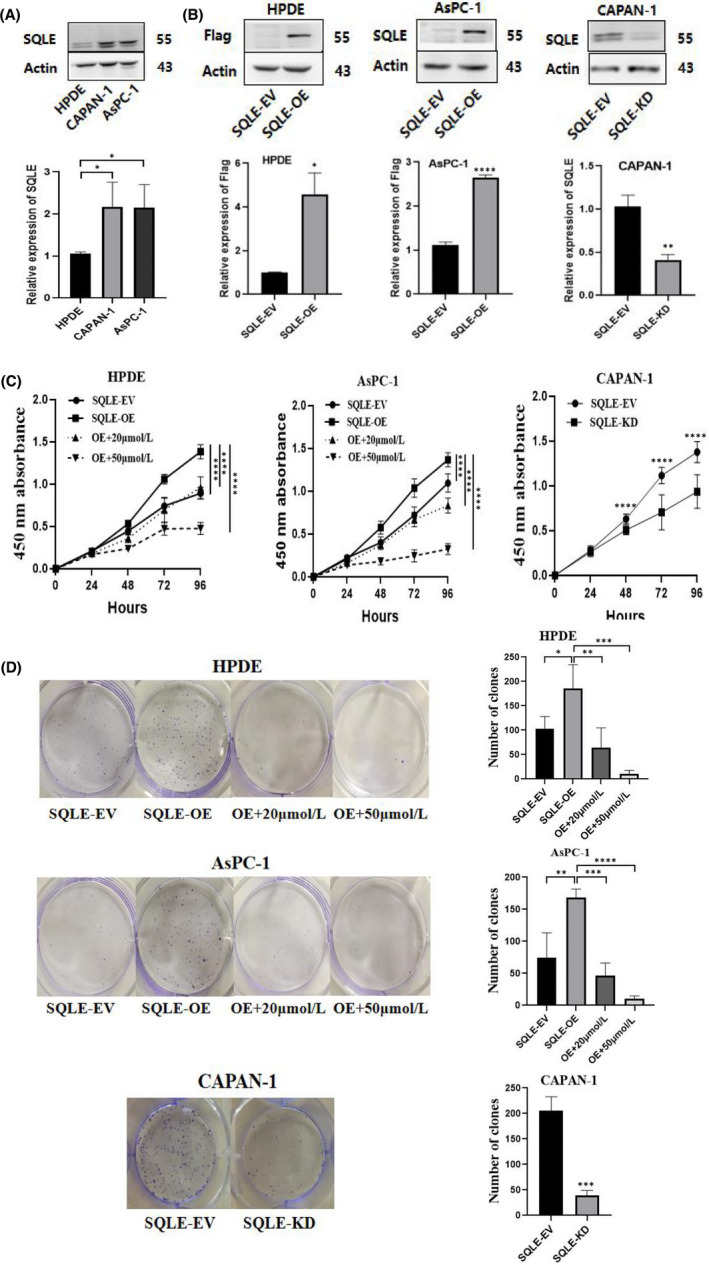
SQLE promotes PC cells proliferation in vitro. (A) SQLE protein levels in two PC cell lines and one normal pancreatic ductal epithelial cell line. (B) Lentiviral vectors were used to establish stable SQLE overexpression and knockdown cell lines. Western Blotting verified the expression of SQLE. (C) Over‐expression of SQLE in HPDE and AsPC‐1 cells obviously promoted cell growth by CCK‐8 assay. Terbinafine inhibited the proliferative effect of SQLE. The knockdown of SQLE in CAPAN‐1 cells inhibited cell growth. (D) Colony forming assay showed increased colony formation in SQLE‐overexpression HPDE and AsPC‐1 cells compared the control cells. Addition of terbinafine significantly reduced the colony formation ability. SQLE‐knockdown CAPAN‐1 cells decreased colony formation. All the data are expressed as the means  ± SD of three independent experiments. *p  <  0.05, **p  <  0.01, ***p  <  0.001, ****p  <  0.0001

The upregulation of SQLE in PC tissue indicated that SQLE can promote the progression of PC. We investigated the expression of SQLE in two PC cell lines, CAPAN‐1, AsPC‐1 and normal pancreatic ductal epithelial cell line HPDE. The result showed that SQLE were significantly upregulated in CAPAN‐1 and AsPC‐1, while silenced in normal pancreatic ductal epithelial cell line HPDE (Figure 2A). To further study the pathogenesis of PC, in addition to up‐regulating the expression of SQLE in normal pancreatic epithelial cell lines, we also upregulated and down‐regulated the expression of SQLE in pancreatic cancer cell lines to explore the effect of SQLE on the biological behaviour of pancreatic cancer cells. HPDE and AsPC‐1 cell lines were transfected with lentiviral virus to upregulate SQLE expression. Then, we divided the overexpressed cell lines into four groups: SQLE‐EV, SQLE‐OE, OE+20 μmol/L terbinafine and OE+50 μmol/L terbinafine. Conversely, we transfected the SQLE RNAi lentiviral vector into the CAPAN‐1 cell line to down‐regulate the expression of SQLE. Western blotting confirmed the expression changes of SQLE (Figure 2B). CCK‐8 assay showed that the over‐expression of SQLE in HPDE and AsPC‐1 cells significantly promoted cell growth. However, the growth of cells added with terbinafine was inhibited. Moreover, the inhibitory effect of adding 50 μmol/L terbinafine was higher than that of 20μmol/L. In addition, the knockdown of SQLE in CAPAN‐1 cells inhibited cell growth compared with the control group (Figure 2C). Colony forming assay showed that over‐expression of SQLE increased colony formation and colony cell number, while low SQLE level and addition of terbinafine significantly reduced colony formation ability (Figure 2D). It should be mentioned that during the CCK‐8 assay, we found that when we cultured the AsPC‐1 cell line for the same time as the CAPAN‐1 cell line, there was no significant difference between AsPC‐1‐SQLE‐EV and CAPAN‐1‐SQLE‐EV. However, the cells in the AsPC‐1‐SQLE‐OE group were connected into sheets due to excessive proliferation, which made it difficult to count the number of clones. In addition, cells added with 50 μmol/L terbinafine could not form clones and could not be counted. Therefore, after comprehensive consideration, we appropriately shortened the culture time of AsPC‐1 cells compared with CAPAN‐1 cells.

### 3.3 SQLE promotes PC cells migration and invasion in vitro

**FIGURE 3:**
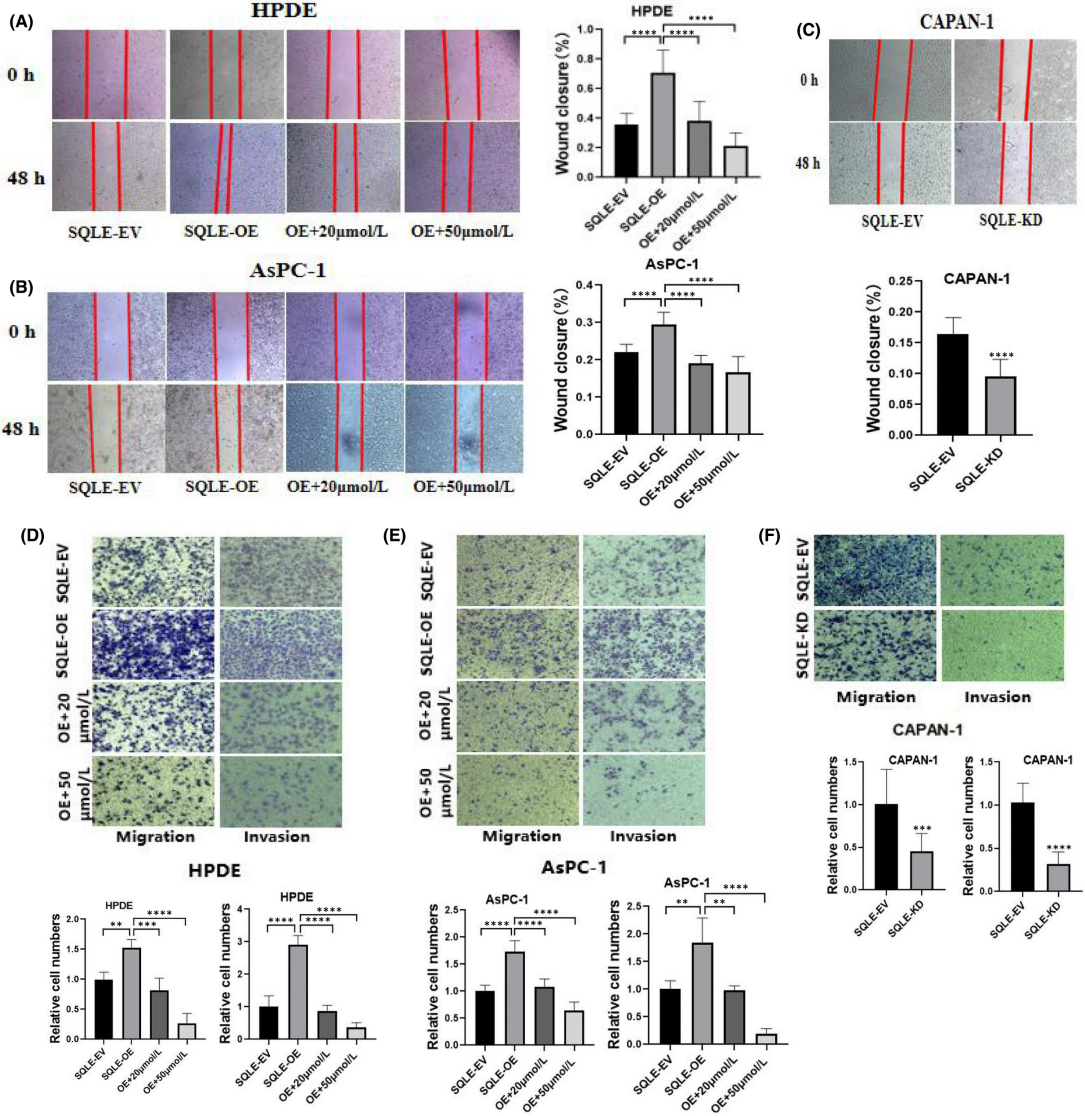
SQLE promotes PC cells migration and invasion in vitro. (A, B, and C) Wound healing assay showed that over‐expression of SQLE in HPDE and AsPC‐1 cells significantly enhanced cell migration. Terbinafine inhibited the migration. The migration was significantly reduced in the SQLE‐knockdown CAPAN‐1 cells. (D, E and F) Cell migration and invasion were detected by transwell assay. All the data are expressed as the means  ± SD of three independent experiments. **p  <  0.01, ***p  <  0.001, ****p  <  0.0001

We further evaluated the migration and invasion ability of SQLE in PC cells. The wound healing assay showed that over‐expression of SQLE in HPDE and AsPC‐1 cells significantly increased cell migration ability. While the addition of terbinafine significantly inhibited the migration, and the inhibitory effect of adding 50 μmol/L terbinafine was higher than that of 20 μmol/L (Figure 3A, B). Compared with the control group, the migration was significantly reduced in the SQLE‐knockdown CAPAN‐1 cells (Figure 3C). Transwell assay showed that the migration and invasion were enhanced in the over‐expression of SQLE in HPDE and AsPC‐1 cells. Terbinafine inhibited the migration and invasion (Figure 3D, E). The migration and invasion were significantly reduced in the SQLE‐knockdown CAPAN‐1 cells compared to the control group (Figure 3F). It is worth mentioning that similar to the CCK‐8 assay, when there was no significant difference between AsPC‐1‐SQLE‐EV and CAPAN‐1‐SQLE‐EV in transwell assay, cells in the AsPC‐1‐SQLE‐OE group were connected into sheets due to the rapid proliferation, resulting in relatively large count error. Therefore, we appropriately shortened the culture time of AsPC‐1 cells compared with CAPAN‐1 cells.

### 3.4 SQLE promotes PC cells proliferation in vivo

**FIGURE 4:**
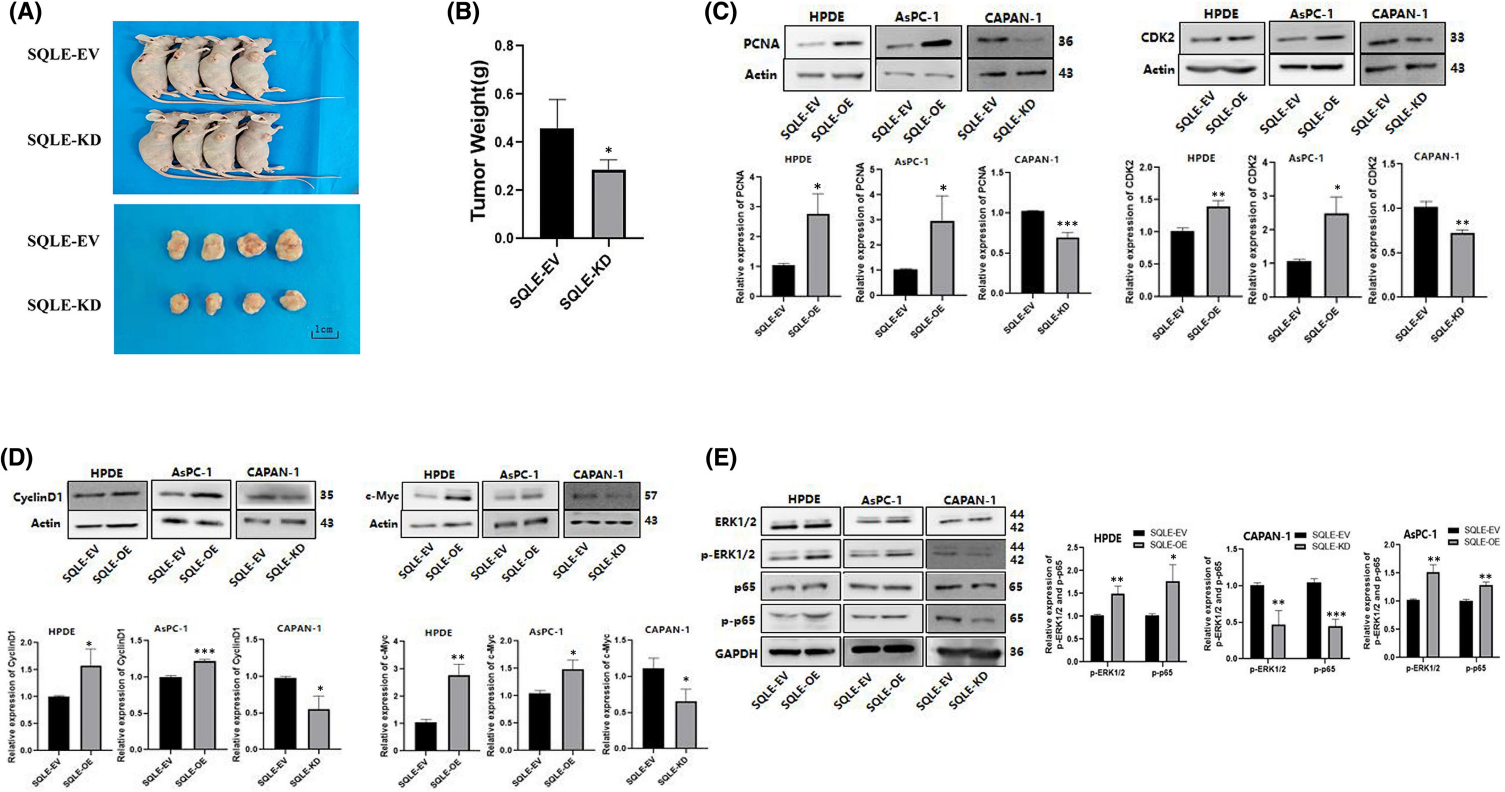
SQLE promotes PC cells proliferation in vivo. Over‐expression of SQLE can promote proliferation and upregulate p‐ERK1/2 and p‐p65 in PC cells. (A) Images of nude mice with xenograft tumours. (B) Weight of xenograft tumours. (C, D) Expression levels of PCNA, CDK2, Cyclin D1, and c‐Myc were detected by Western blotting. (E) Expression levels of p‐ERK1/2 and p‐NF‐κB (p‐p65) were detected by Western blotting. All the data are expressed as the means  ± SD of three independent experiments. *p  <  0.05, **p  <  0.01, ***p  <  0.001

We chose the CAPAN‐1 cell line transfected with SQLE RNAi lentivirus to explore the tumorigenic ability of SQLE in vivo. Nude mouse tumorigenesis assay showed that the tumour weight of nude mice injected with SQLE‐knockdown CAPAN‐1 cells reduced significantly compared with the control group (Figure 4A, B).

### 3.5 Over‐expression of SQLE can promote proliferation and upregulate p‐ERK1/2 and p‐ NF‐κB in PC cells

To further explore the role of SQLE in the proliferation of PC cells, we tested the expression levels of proliferation‐related proteins. The results showed that compared with the control group, the expressions of PCNA, CDK2, Cyclin D1 and c‐Myc protein in the SQLE overexpression groups were significantly increased. Moreover, the expression of SQLE‐knockdown group was significantly reduced (Figure 4C, D).

In addition, we examined the changes in ERK and NF‐κB and their phosphorylation levels after up‐regulation and down‐regulation of SQLE expression to explore the potential molecular mechanism of SQLE in promoting the progression of PC. The results showed that p‐ERK1/2 and p‐NF‐κB (p‐p65) were upregulated in the SQLE‐overexpression HPDE and AsPC‐1 cells, while the expression of these proteins showed the opposite trend in SQLE‐knockdown CAPAN‐1 cells (Figure 4E).

### 3.6 MiR‐133b is down‐regulated in PC tissues and negatively correlated with the expression of SQLE

**FIGURE 5:**
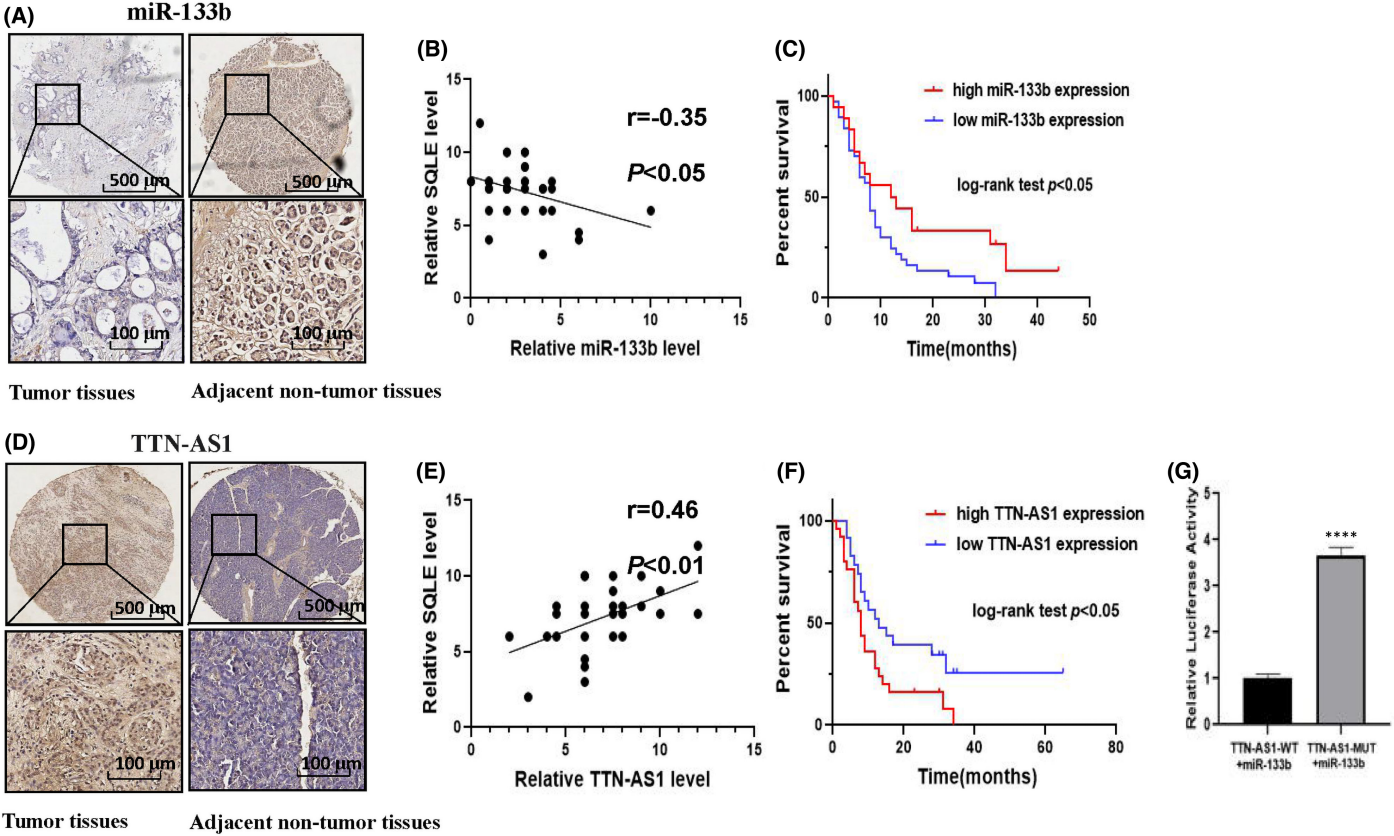
MiR‐133b is down‐regulated in PC tissues and negatively correlated with the expression of SQLE. LncRNA‐TTN‐AS1 regulates miR‐133b as a ceRNA and is highly expressed in PC. (A) Representative images of ISH staining of miR‐133b in PC tissues and adjacent tissues. (B) Correlation of miR‐133b and SQLE levels in PC patients. (C) Survival curves of PC patients with high and low miR‐133b expression. (D) Representative images of ISH staining of LncRNA‐TTN‐AS1 in PC tissues and adjacent tissues. (E) Correlation of LncRNA‐TTN‐AS1 and SQLE levels in PC patients. (F) Survival curves of PC patients with high and low LncRNA‐TTN‐AS1 expression. (G) The luciferase intensity was decreased by cotransfected miR‐133b mimics and lncRNA‐TTN‐AS1‐WT but not in the mutant reporter vector lacking the putative miR‐133b binding site. ****p  <  0.0001

It is well known that miRNAs can participate in tumour progression by binding to target genes. In order to explore the potential upstream mechanism that SQLE promotes the development of PC, we used the target prediction tool TargetScan to make predictions. The results showed that miR‐133b was a direct upstream target of SQLE (Figure S1). In addition, studies have confirmed that miR‐133b suppressed the expression of SQLE by directly targeting the SQLE 3′‐UTR in oesophageal squamous cell carcinoma. Therefore, we speculate that miR‐133b is likely to inhibit the expression of SQLE by targeting the SQLE 3'‐UTR in pancreatic cancer, thereby further inhibiting the development of pancreatic cancer. We tested the expression level of miR‐133b in PC tissue microarray. The results demonstrated that miR‐133b was down‐regulated in PC tissues (Figure 5A) and negatively correlated with the expression of SQLE (Figure 5B). Moreover, the prognosis of PC patients with low expression of miR‐133 was even worse (Figure 5C).

### 3.7 LncRNA‐TTN‐AS1 regulates miR‐133b as a ceRNA and is highly expressed in PC

Studies have confirmed that non‐coding RNAs as competing endogenous RNA (ceRNA) bind to miRNAs and protect their target RNAs from repression or degradation. In order to find lncRNAs targeting miR‐133b, we use LncBase Predicted to make predictions. The results show that lncRNA‐TTN‐AS1 is a miR‐133b–targeting lncRNA (Figure S2). So, we speculate that TTN‐AS1 may participate in the progression of PC by regulating the expression of miR‐133b. We measured the expression levels of lncRNA‐TTN‐AS1 in PC tissues and adjacent normal tissues by ISH assay. The results demonstrated that lncRNA‐TTN‐AS1 was mainly expressed in the PC tissues (Figure 5D) and positively correlated with the expression of SQLE (Figure 5E). Kaplan–Meier analysis showed that PC patients with overexpression of TTN‐AS1 have a worse prognosis than those with low expression of TTN‐AS1 (Figure 5F). In addition, we performed luciferase reporter assay to test the direct binding between lncRNA‐TTN‐AS1 and miR‐133b. The results illustrated that transfection with miR‐133b could obviously inhibit luciferase activity driven by the wild‐type lncRNA‐TTN‐AS1 3′‐UTR, while the mutant lncRNA‐TTN‐AS1 3′‐UTR could not be inhibited (Figure 5G). In summary, these data suggested that lncRNA‐TTN‐AS1 regulates miR‐133b as a ceRNA to participate in the progression of PC.

## 4 DISCUSSION

Previous reports have shown that the accumulation of SQLE can be observed in a variety of tumours. However, the biological role of SQLE in the development of PC is unclear. In this study, we first demonstrated that SQLE significantly promoted the progression and metastasis of PC. In addition, terbinafine, the SQLE inhibitor, inhibited the proliferation and metastasis of PC cells in cell culture. Moreover, we further explored the upstream molecules that may regulate SQLE to study its carcinogenic mechanism.

Extracellular signal regulated kinase (ERK) is an important part of mitogen‐activated protein kinase (MAPK), which regulates cell proliferation, differentiation and survival. ERK and its signalling pathway act on nuclear transcription factors such as AP‐1 and NF‐к B through ERK signalling cascade to regulate gene expression. An increasing body of evidence suggests that ERK/NF‐κB pathway plays an important role in many cancers.^22^, ^23^, ^24^, ^25^ In this study, we elucidated that p‐ERK1/2, p‐NF‐κB (p‐p65) were upregulated in the SQLE‐overexpression cells, while showed the opposite trend in SQLE‐knockdown cells. These data suggested that the effect of SQLE on PC may be related to ERK/NF‐κB pathway.

In order to further study the role of SQLE in the PC process, we focused on its upstream regulators. MicroRNAs (miRNAs) can regulate the expression level of specific proteins according to sequence complementarities with their target mRNA molecules, and they play crucial roles in the tumorigenesis and development.^26^ MiR‐133b has been identified as a tumour suppressor gene in a variety of cancers, and is closely related to suppressed tumour metastasis.^27^ However, the expression and role of miR‐133b in PC have not been reported. Predicted by the public database TargetScan, we found that miR‐133b may interact with SQLE. In addition, studies have confirmed that miR‐133b inhibits the expression of SQLE by binding to the 3'‐UTR of SQLE.^9^ Therefore, we speculated that miR‐133b might regulate the ERK/NF‐κB pathway by acting on SQLE, thereby participating in the progression of pancreatic cancer. Our research showed that the expression of miR‐133b in PC tissues was significantly down‐regulated and negatively correlated with the expression of SQLE. Moreover, PC patients with suppression of miR‐133b had a worse prognosis. These showed that SQLE was negatively regulated by miR‐133b, which had a certain impact on cell proliferation, migration and invasion of PC.

Recently, it has been found that some lncRNAs act as competitive endogenous RNAs (ceRNAs) by binding to miRNAs (‘sponging’) and reducing their inhibition on targets.^28^ The abnormal expression of lncRNAs has been proved to be related to the development of cancer.^29^, ^30^ In order to further clarify the upstream regulatory pathway of miR‐133b, we used LncBase Predicted to predict, and the results showed that lncRNA‐TTN‐AS1 could target miR‐133b (Figure S2). Therefore, we speculated that lncRNA‐TTN‐AS1 could regulate the expression level of SQLE by sponging miR‐133b, and then acted on the ERK/NF‐κB pathway to affect the progression of pancreatic cancer. Our research results showed that lncRNA‐TTN‐AS1 was mainly expressed in the PC tissues and positively correlated with the expression of SQLE. In addition, its high expression was associated with poor prognosis in PC patients. Moreover, Luciferase reporter gene detection showed that lncRNA‐TTN‐AS1 could directly bind to miR‐133b. These data suggested that lncRNA‐TTN‐AS1 regulated miR‐133b as a ceRNA to participate in the progression of PC.

Lately, a study has shown that SQLE could involved in maintaining the stemness of breast cancer stem cells and breast tumour progression by enhancing cholesterol synthesis.^31^ In addition, Jun SY et al found that SQLE reduction caused by cholesterol accumulation aggravates CRC progression via the activation of the β‐catenin oncogenic pathway and deactivation of the p53 tumour suppressor pathway.^32^ These studies led us to speculate whether SQLE and terbinafine are involved in pancreatic cancer progression by regulating the expression of cholesterol. Does excess cholesterol in turn regulate the expression of SQLE to affect the progression of pancreatic cancer? These are questions we need to explore further in the future.

**FIGURE 6:**
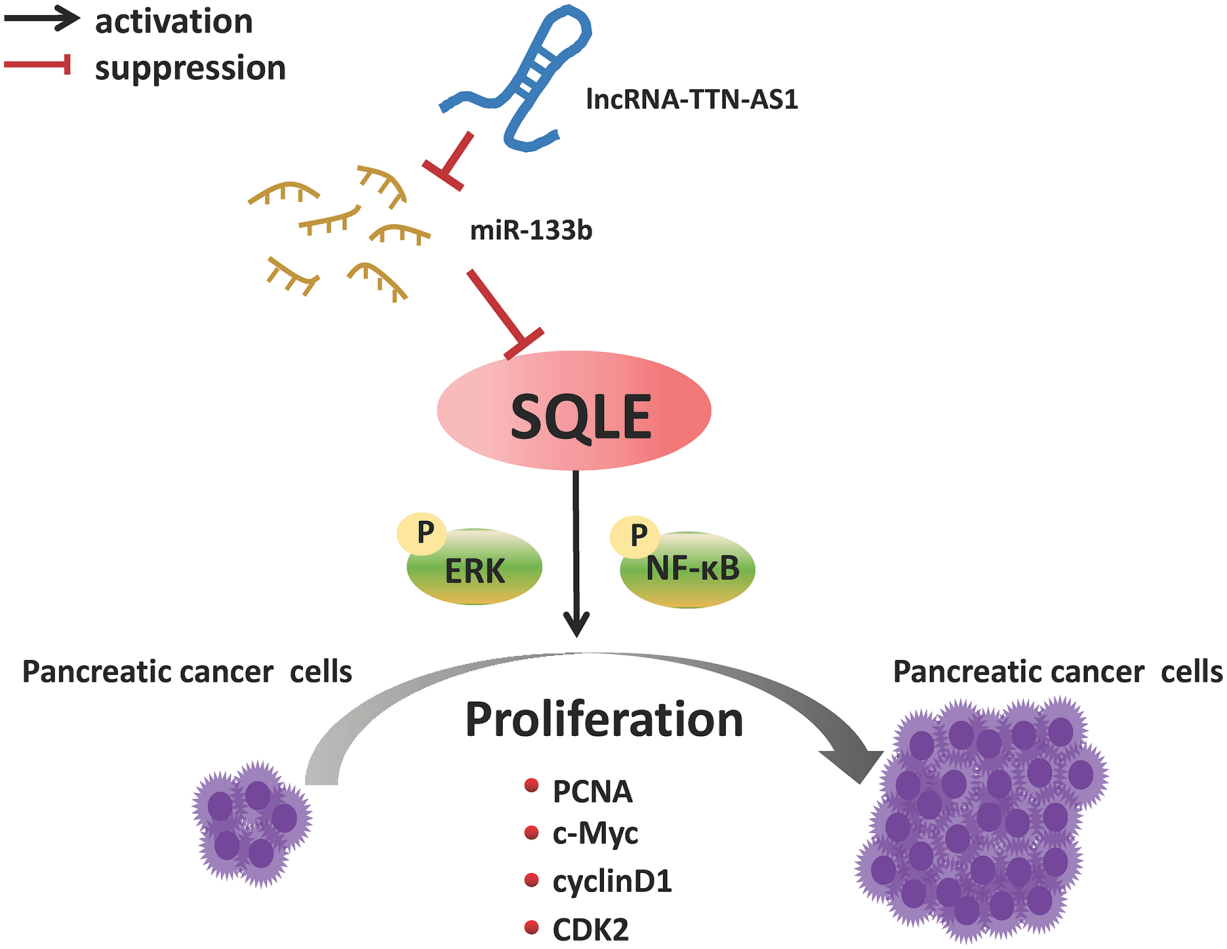
Molecular mechanism of SQLE in PC progression. LncRNA‐TTN‐AS1 can act as a molecular sponge of miR‐133b to regulate SQLE expression, and high levels of SQLE can promote pancreatic cancer proliferation and progression by activating ERK/NF‐κB signalling pathway

In conclusion, our study supports that lncRNA‐TTN‐AS1 can act as a molecular sponge of miR‐133b to regulate SQLE expression, and high levels of SQLE can promote pancreatic cancer proliferation and progression by activating ERK/NF‐κB signalling pathway (Figure 6). Targeting the lncRNA‐TTN‐AS1/miR‐133b/SQLE axis is expected to provide new ideas for the clinical treatment of PC patients.

## AUTHOR CONTRIBUTION

**Shuhui Wang:** Data curation (lead); Investigation (lead); Writing – original draft (lead). **Lei Dong:** Project administration (lead); Supervision (lead); Writing – original draft (lead). **Lin Ma:** Methodology (equal); Writing – original draft (supporting). **Suzhen Yang:** Validation (equal); Writing – original draft (supporting). **Ying Zheng:** Software (equal); Writing – original draft (supporting). **Jing Zhang:** Methodology (equal); Supervision (supporting). **Chuanghong Wu:** Investigation (supporting); Supervision (supporting). **Yidi Zhao:** Data curation (supporting); Formal analysis (supporting). **Yangfan Hou:** Data curation (supporting). **Hong Li:** Conceptualization (equal); Project administration (equal); Supervision (equal). **Ting Wang:** Conceptualization (lead); Data curation (equal); Project administration (equal); Supervision (equal).

## CONFLICT OF INTEREST

The authors have no conflict of interest.

## Supporting information

Fig S1

Fig S2

## Data Availability

The data that support the findings of this study are available from the corresponding author upon reasonable request.
