# Supplementary figures and images for: SQLE facilitates the pancreatic cancer progression via the lncRNA‐TTN‐AS1/miR‐133b/SQLE axis
Source: J Cell Mol Med. 2022 May 31;26(13):3636–47. doi: 10.1111/jcmm.17347 (PMC9258714; doi:10.1111/jcmm.17347)

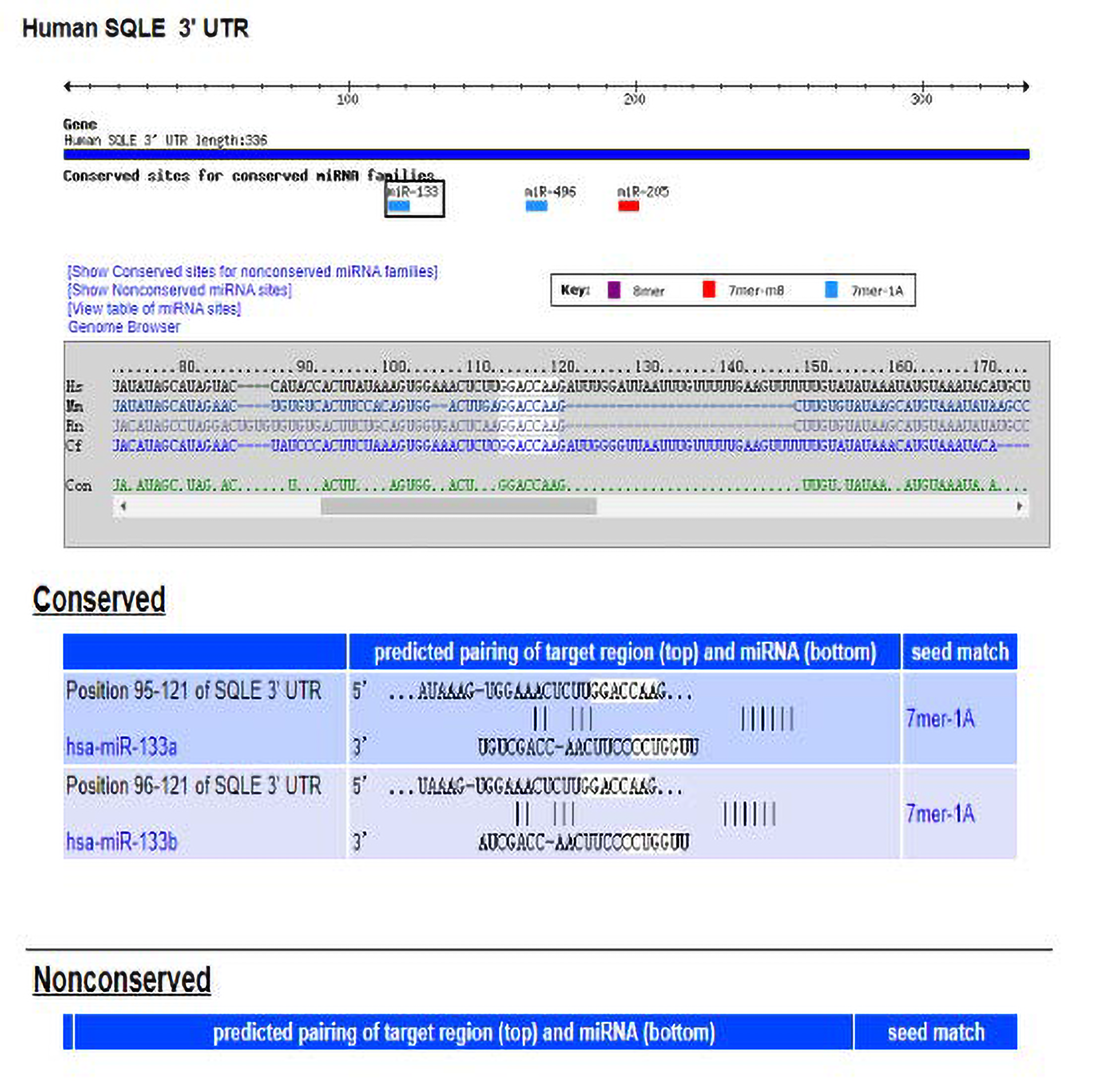

Supplement: Supplementary file 1 — Fig S1 [file JCMM-26-3636-s002.tif]

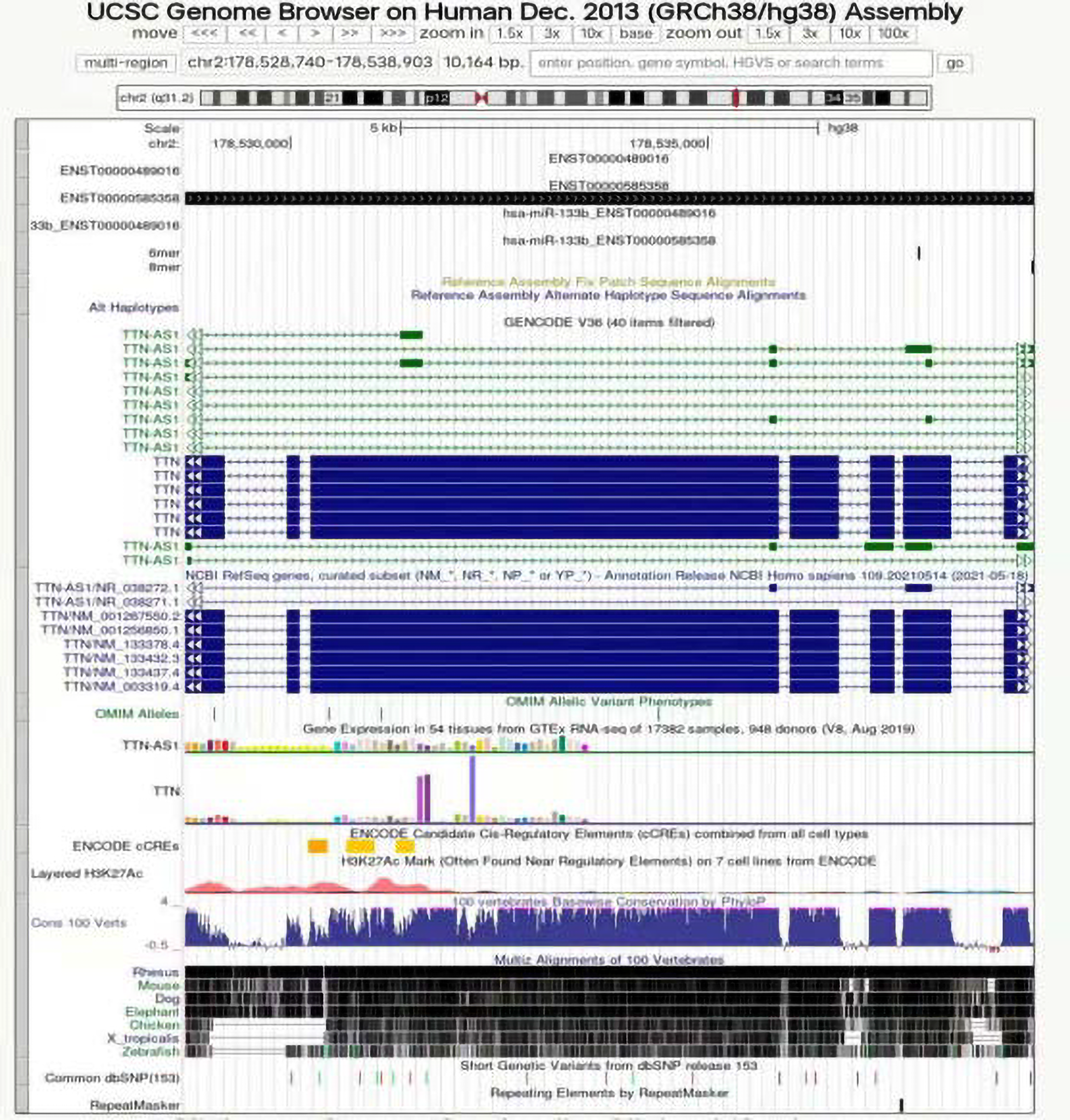

Supplement: Supplementary file 2 — Fig S2 [file JCMM-26-3636-s001.tif]
